# Increased reports of severe myocarditis associated with enterovirus infection in neonates, United Kingdom, 27 June 2022 to 26 April 2023

**DOI:** 10.2807/1560-7917.ES.2023.28.39.2300313

**Published:** 2023-09-28

**Authors:** Anika Singanayagam, Catherine Moore, Susannah Froude, Cristina Celma, Julia Stowe, Erjola Hani, Khuen Foong Ng, Peter Muir, Marion Roderick, Simon Cottrell, David F. Bibby, Barry Vipond, Sophie Gillett, Peter J. Davis, Jack Gibb, Mai Barry, Phillippa Harris, Frances Rowley, Jiao Song, Ananda Giri Shankar, Danielle McMichael, Jonathan M. Cohen, Abirami Manian, Ciaran Harvey, Louise Shaw Primrose, Stefanie Wilson, Declan T. Bradley, Karthik Paranthaman, Stuart Beard, Maria Zambon, Mary Ramsay, Vanessa Saliba, Shamez Ladhani, Christopher Williams

**Affiliations:** 1 UK Health Security Agency, Colindale, London, United Kingdom; 2 Public Health Wales, Wales, Cardiff, United Kingdom; 3 Paediatric Infectious Diseases and Immunology, Bristol Royal Hospital for Children, Bristol, United Kingdom; 4 UK Health Security Agency South West Regional Laboratory, Bristol, United Kingdom; 5 Paediatric Intensive Care Unit, Bristol Royal Hospital for Children, University Hospitals Bristol & Weston Foundation Trust, Bristol, United Kingdom; 6 Department of Paediatric Cardiology, Bristol Royal Hospital for Children, University Hospitals Bristol & Weston Foundation Trust, Bristol, United Kingdom; 7 Public Health Agency, Belfast, United Kingdom; 8 Evelina London Children’s Hospital, Guys & St Thomas National Health Service Foundation Trust, London, United Kingdom; 9 Public Health Scotland, Glasgow, United Kingdom

**Keywords:** neonatal infection, enterovirus, coxsackie, myocarditis

## Abstract

Enteroviruses are a common cause of seasonal childhood infections. The vast majority of enterovirus infections are mild and self-limiting, although neonates can sometimes develop severe disease. Myocarditis is a rare complication of enterovirus infection. Between June 2022 and April 2023, twenty cases of severe neonatal enteroviral myocarditis caused by coxsackie B viruses were reported in the United Kingdom. Sixteen required critical care support and two died. Enterovirus PCR on whole blood was the most sensitive diagnostic test. We describe the initial public health investigation into this cluster and aim to raise awareness among paediatricians, laboratories and public health specialists.

**Key public health message**

**What did you want to address in this study?**

Inflammation of the heart muscle (myocarditis) is a rare but serious complication of enterovirus (EV) infection. After clinicians in south Wales and the south-west of England noted an unusually high number of cases of myocarditis in newborn babies linked to coxsackie B viruses (a type of EV) in 2022/23, we undertook a clinical and public health investigation.

**What have we learnt from this study?**

Newborn babies with neonatal enteroviral myocarditis (NEM) were severely unwell, with shock as the presenting feature of myocarditis, often requiring critical care support for prolonged periods. Using PCR on whole blood was the most sensitive sample for confirming EV infection. The upsurge of NEM cases overlapped with high seasonal EV activity and increased circulation of coxsackie-B3 and coxsackie-B4 viruses.

**What are the implications of your finding for public health?**

Occasionally, outbreaks of severe disease caused by specific EV serotypes may occur. Clinical awareness and harmonised surveillance (epidemiological/clinical/virological) for severe EV infection is important for identifying and characterising these outbreaks. Cases of NEM should be reported to relevant public health bodies and positive samples submitted to reference laboratories for confirmation, serotyping and further characterisation.

## Background

Non-polio enteroviruses (NPEV) exhibit seasonal patterns of incidence, with frequent fluctuations in predominating enterovirus (EV) serotypes. In Europe, 50–65% of infections affecting infants occur between April and October [1]. The vast majority of EV infections are mild and self-limiting. Occasionally, increased reports of severe disease caused by specific EV serotypes are noted. For example, in 2018 increased reports of acute flaccid paralysis mostly in children under 5 years were associated with an increase in EV-D68 detections in the United Kingdom (UK) [2]. Other reports across Europe have included an upsurge of echovirus 30 infections associated with meningoencephalitis in 2018 [3] and neonatal sepsis associated with echovirus 11 in 2023 [4]. However, as systematic surveillance for NPEVs is limited, and NPEV infection is not a notifiable disease in many countries, severe disease outbreaks may only be identified opportunistically, and evidence is lacking on the true burden of disease.

Severe clinical presentations of NPEV infection can occur in the neonatal period including sepsis, meningoencephalitis, hepatitis, coagulopathy and pneumonia [5]. Myocarditis is a rare but well-described complication of NPEV infection, in particular the coxsackie B viruses, and associated with severe long-term complications and death across all age groups, including children [6] and, specifically, neonates [1,7-10], with a case fatality rate of 30–50% [9]. Here, we report on an upsurge of severe EV myocarditis in neonates in the UK and the subsequent epidemiological and virological investigations.

## Outbreak detection

On 5 April 2023, Public Health Wales notified the UK Health Security Agency (UKHSA) of 10 neonatal enteroviral myocarditis (NEM) cases presenting between June 2022 and April 2023. The cluster had been alerted by clinicians concerned about an unusual number of cases. On 28 April 2023, UKHSA led a joint incident management meeting to investigate this cluster in the four UK nations England, Northern Ireland, Scotland and Wales. Five additional cases were concurrently reported by clinicians from south-west England, a region in geographical proximity to south Wales, where a tertiary level paediatric cardiology centre is located. Retrospective case notes review had identified only two NEM cases across south Wales and south-west England in the 6 years before June 2022 (one case each in March 2022 and October 2021), suggesting an unusual increase in case numbers had been observed in this geographical region. Active case finding, through communications to clinical networks and microbiology, subsequently identified two further NEM cases in Scotland and three in England over the same period. No cases were identified in Northern Ireland.

**Figure 1:**
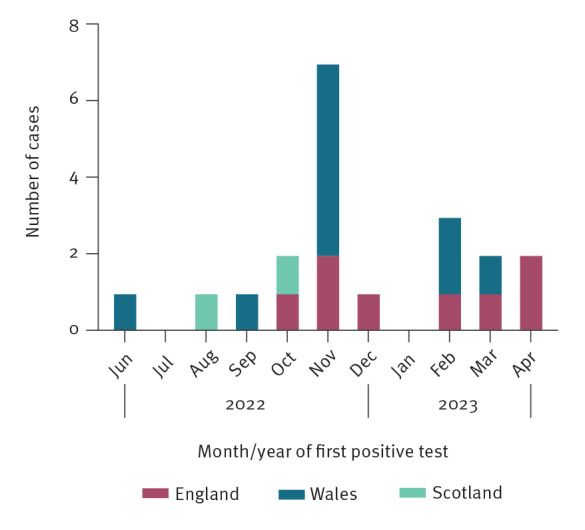
Epidemiological curve of neonatal (< 28 days) enteroviral myocarditis cases, United Kingdom, 27 June 2022–26 April 2023 (n = 20)

Neonates presented to hospital severely unwell, with shock as the presenting feature of myocarditis, and required ventilatory and circulatory support in paediatric intensive care unit (PICU), often for prolonged periods. All 20 NEM cases were hospitalised, with 16 admitted to critical care and two died. Median duration of PICU stay was 33 days (interquartile range 13–47 days). One case died before admission to PICU. The cluster peaked in November 2022 (seven cases), with sporadic cases in other months (Figure 1).

**Figure 2:**
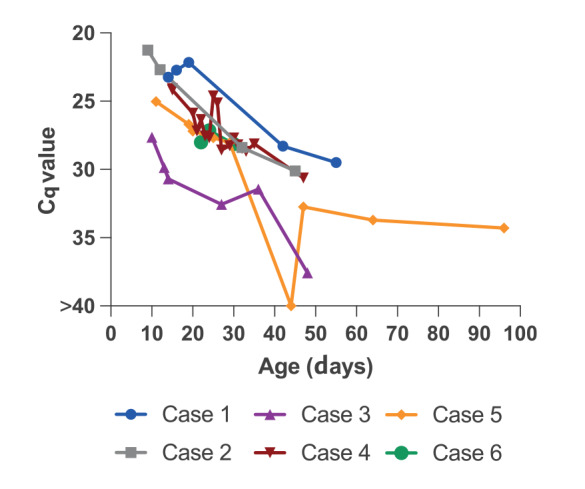
Enterovirus PCR quantification cycle (Cq) values on whole blood specimens from six neonatal (< 28 days) enteroviral myocarditis cases, where sequential sampling was available, United Kingdom

Median age at EV diagnosis was 10 days (interquartile range 8–11 days); nine of 20 were female. Fifteen of 18 neonates with known gestation had been born at term. Of 17 EV strains that were typed, nine were coxsackie B3 (CVB3), six were coxsackie B4 (CVB4), one was coxsackie B5 (from Scotland) and one was coxsackie B1 (from England). Where sampled, EV PCR on whole blood (16/16 sampled) was most frequently positive, followed by stool (10/17), then upper respiratory tract (nose swab/throat swab/nasopharyngeal aspirate) (8/18). Eight had a lumbar puncture and seven tested EV-positive in cerebrospinal fluid (CSF). More details can be seen in Supplementary Table 1. Where sequential blood EV PCR was available (≥ 2 samples on different days), for six of the cases, EV detection in blood persisted over prolonged periods of follow up (median 37 days (range 8–85 days)) (Figure 2). All 17 delivering mothers in whom vaccination records were available had received prior COVID-19 vaccination, with three of 17 vaccinated during pregnancy, in line with overall national vaccination uptake rates. Maternal immunisation with a COVID-19 vaccine during pregnancy is therefore unlikely to be associated with NEM in our cohort. Data on maternal EV status were not available. The PICUs in south-west England (three cases) and Wales (five cases) published a case series on eight of the NEM cases diagnosed between September and December 2022 [11], describing the clinical features and management.

Cq: quantification cycle.

Samples with a Cq value > 40 were considered negative.

## Methods

### Case definition

Cases of NEM were defined as neonates (aged < 28 days) with a positive EV sample, with clinical or post-mortem diagnosis of myocarditis. These cases were reported by clinicians to public health authorities via case reporting forms, encouraged through communications to clinical and microbiology networks. Contacts, including parents, were not routinely screened for recent enterovirus infection. The source of infection for the neonates could have been within or outside the household and any recent infections in contacts may not be detectable by PCR testing due to timing of presentation.

### Data sources

#### Second Generation Surveillance System (SGSS)

In England, detections of EV are reported voluntarily by hospital laboratories to UKHSA via the Second-Generation Surveillance System (SGSS) electronic database. We extracted data for all EV positive reports between 1 January 2003 and 7 May 2023. More than one sample may have been collected per patient.

#### Hospital Episode Statistics

In England, infants aged 0–90 days with a discharge diagnosis of acute myocarditis International Classification of Diseases (ICD-10) code I40 or unspecified myocarditis I514 or myocarditis in viral disease classified elsewhere I411 were identified, by month of year and age group [12,13]. The age of the infant was the age when a myocarditis ICD-10 code was first recorded in Hospital Episode Statistics (HES), rather than the age at symptom onset or age at hospitalisation.

#### Patient Episode Database for Wales

Infants aged 0–90 days with a discharge diagnosis of acute myocarditis ICD-10 I40 or unspecified myocarditis I514 or myocarditis in viral disease classified elsewhere I411 were identified, by month of year and age group. The age of the infant was calculated as age at admission date.

### Enterovirus PCR on whole blood – sequential testing

Cases that had sequential testing of EV PCR on whole blood (Figure 2) were tested at UKHSA South West Regional Laboratory. Total nucleic acid was extracted from blood samples using a Blood DNA minikit on a Qiasymphony robot (Qiagen). Enterovirus RNA was detected by two-step reverse transcription-real-time PCR using previously described primers and probes [14].

### Enterovirus typing

Isolates of EV were genotyped at the Enteric Virus Unit, UKHSA Colindale, using the method described elsewhere [15]. Purified DNA products were sequenced using an ABI Prism 3130 genetic analyzer (Applied Biosystems). Genotype was assigned by comparison with sequences available in public databases using the Dutch National Institute for Public Health and the Environment (RIVM) and the Basic Local Alignment Search Tool (BLAST) online sequence analysis tools [16]. Sequences were deposited in GenBank (OR340791-OR340831).

### Phylogenetic analysis

The UKHSA CV-B3 and CV-B4 partial VP1 sequence datasets were aligned to 40 and 18 reference genomes, and 142 and 59 surveillance sequences, respectively, using mafft v7.490 [17]. The CV-B3 alignment was trimmed to nucleotide (nt) positions 2562–2870 of strain Nancy (M33854), and the CV-B4 alignment to nt positions 2558–2885 of strain JVB (X05690). Maximum likelihood trees were generated from the trimmed alignments using IQTree2 [18] with 1000 bootstrap replicates. A generalised time reversible (GTR) model with six rate categories was specified, along with a 500-iteration stopping parameter. Guide trees generated by FastTree2 [19] were passed as starting topologies. The CV-B3 and CV-B4 algorithms were given random number seeds of 656016 and 497398, respectively. Figures were annotated using the Interactive Tree of Life iTol v6.8 (itol.embl.de).

## Results

### Epidemiological investigations

#### Enterovirus circulation

We reviewed national trends in EV circulation. Data from England and Wales are considered separate due to differences in testing and data collection.

**Figure 3:**
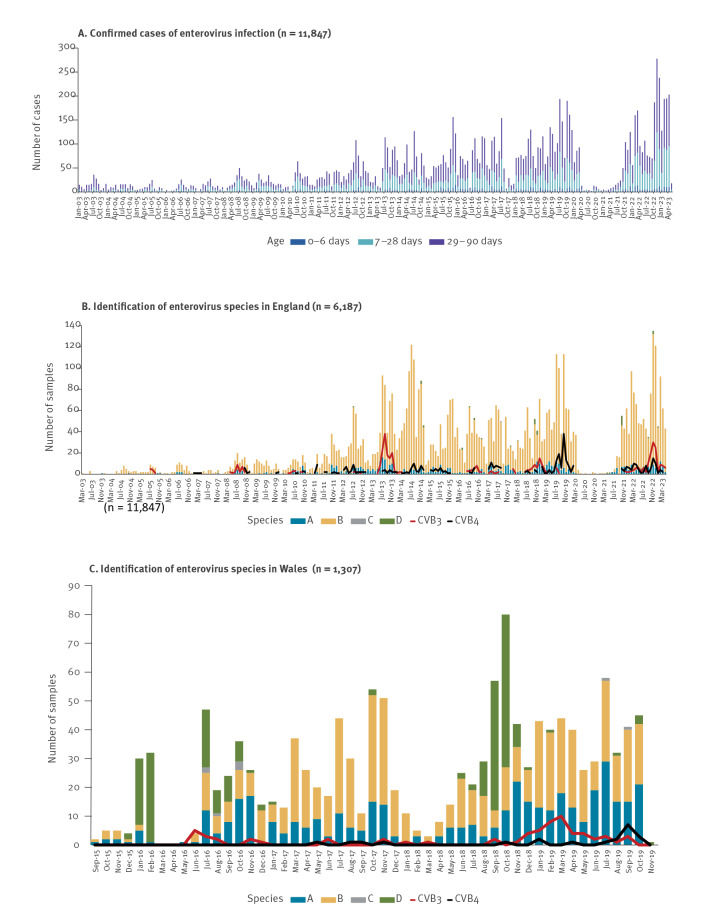
Detection and identification of enterovirus in infants aged < 90 days, England and Wales, 2003–2023

CVB: coxsackie B virus; SGSS: Second-Generation Surveillance System.

In England (population ca 56.5 million, ca 595,000 annual births [20]), hospitals provide PCR-testing for EV based on clinician request, though testing practices and assays vary. Positive results (including screenings for respiratory pathogens that sometimes do not differentiate between EV and rhinovirus) are recorded in the SGSS on a voluntary basis. Hospital laboratories are recommended to submit all confirmed samples, especially from patients with severe EV infections and acute flaccid paralysis, to the national reference laboratory for typing, but uptake is variable, with < 40% of confirmed samples submitted for typing. Tests for EV that are performed but are negative are not nationally reported. In SGSS increasing reports of EV infections since 2003 have been seen as more hospitals implemented PCR testing for EV [21]. Enteroviral infections show a characteristic seasonal pattern, peaking in late summer or early autumn. After very limited circulation of EVs in 2020/2021, likely due to decreased viral transmission associated with COVID-19 pandemic mitigations [22], several reports of EV were received in autumn 2021/2022 and 2022/2023. This is shown in Supplementary Figure 1. Confirmations of EV in infants aged < 90 days (Figure 3A) typically followed the same pattern as total confirmations across all ages, shown in Supplementary Figure 1. Enterovirus species B was the most prevalent among typed isolates from infants aged < 90 days. Enteroviral species CVB3 and CVB4 have been detected throughout the surveillance period (since 2003), with upsurges in 2013, 2018 and 2022 for CVB3 and in 2019 and 2022 for CVB4 (Figure 3B).

Numbers of confirmed cases were retrieved from SGSS or from the national reference laboratory and numbers of species identifications were obtained from the national reference laboratory.

Number of samples typed as coxsackie virus B3 (CVB3) and coxsackie virus B4 (CVB4) are depicted in red and black lines, respectively.

In Wales (population ca 3 million, ca 28,000 annual births [20]), positive samples from patients with symptoms consistent with EV infection and/or respiratory symptoms are tested by a specific EV assay with typing of positives (enabling differentiation of enterovirus and rhinovirus). Surveillance for EV has been active in Wales since 2015, with a pause over the pandemic [23]. Data are collected on all EV tests performed (positive and negative). The proportion of EV-positive tests did not significantly increase in 2022/23 which can be seen in Supplementary Figure 2. Between 2015 and 2019, as part of a pilot programme, EV typing was attempted on all EV-positive samples. An increase in EV detections in infants aged < 90 days was noted in 2018/2019, associated with peaks in CVB3 and CVB4 (Figure 3C), in line with trends observed in England.

#### Myocarditis hospitalisations

**Figure 4:**
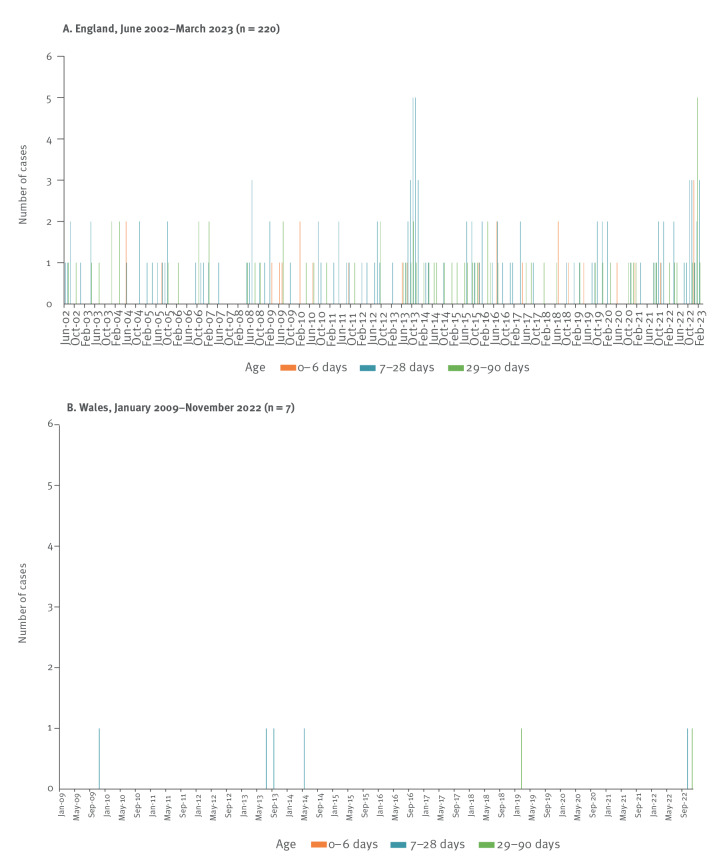
Hospitalisations of myocarditis cases in infants (aged < 90 days) in England and Wales, 2002–2023

Additionally, we sought to investigate national trends in myocarditis hospitalisations by using the HES database for England and the Patient Episode Database for Wales (PEDW). Hospitalised infants aged < 90 days with myocarditis (which may include EV and non-EV causes) between 2003 and 2023 were identified (Figure 4) [12]. In data from England (Figure 4A), peaks in myocarditis hospitalisations in 2013 and 2022 were noted. These were temporally associated with increased CVB3 activity (2013) or increased CVB3 and CVB4 activity (2022) (Figure 3B). There was no evidence of geographic clustering in the 2013 peak - 22 myocarditis cases (diagnosed between September 2013 and March 2014) were admitted to 11 different hospitals, with 0–3 cases per hospital. Hospitals with three cases were all tertiary referral centres with PICUs. There was also no evidence of geographical clustering in the 2022 myocarditis peak - 20 myocarditis cases were admitted to 14 hospitals between October 2022 and February 2023. In Wales, a small peak in myocarditis hospitalisation was also seen in 2013, in line with that observed in England, along with some of the recent 2022 cases (Figure 4B). A reporting lag means that not all cases from the 2022 cluster are plotted.

Data on hospital admissions in England were retrieved from the Hospital Episode Statistics (HES) database and in Wales from the Patient Episode Database for Wales (PEDW).

### Virological investigations

Partial VP1 sequencing was performed for EV assignment on 52 samples from 11 cases at UKHSA. Phylogenetic analysis of available cases sequences (CVB3 n = 23; CVB4 n = 15) did not identify any segregation of NEM cases compared with other UK surveillance materials (CVB3 n = 142; CVB4 n = 59 samples). More detail can be seen in Supplementary Figures 3a and 3b.

### Outbreak control measures

Following incident management meetings on 26 April (Wales) and 28 April (UKHSA), UKHSA established a national routine incident response on 2 May 2023 to coordinate NEM case finding across the UK. The UK Health Security Agency communicated with World Health Organization (WHO) and European Centre for Disease Prevention and Control (ECDC) to alert other countries; public communications were circulated by the WHO [24]. No other country reported NEM clusters. Briefing notes were issued to National Health Service (NHS) partners, paediatric and neonatal networks, to raise awareness and guide reporting and testing approaches. Clinicians were requested to notify NEM cases to UKHSA [25]. Retrospective and prospective clinical reporting is ongoing. Further investigation, including of possible maternal factors, is also ongoing.

## Discussion

We report an increase in NEM from the second half of 2022, associated with high seasonal NPEV activity and increased circulation of CVB3 and CVB4. Cases were clustered in south Wales and south-west England. This clustering is unusual but appears consistent with a national increase in CVB3 and CVB4 circulation although, notably, no other contemporaneous clusters were identified across the rest of the UK. Interestingly, the same region (south-west England) reported 30 children with severe EV infection, including eight with myocarditis due to coxsackie B virus, during 2012/13 [26]. Again, a historic look at national surveillance data indicated a surge in CVB3 detections and also a peak in neonatal myocarditis hospitalisations in 2013. Reasons for possible regional clustering in 2013 and then again in 2022 are not clear. We did not identify any evidence through phylogenetic analysis that NEM cases in this cluster were genetically different from CVB3/CVB4 viruses circulating in the UK, although the analysis was limited to partial VP1 sequencing of available samples (those sent to the reference laboratory). High seasonal EV activity in 2022/23 may relate to reduced exposure over the COVID lockdown period, perhaps increasing susceptibility and/or severity in neonates of unexposed mothers.

As EV infection is not among notifiable diseases for many countries, severe infections could go unreported [27]. Moreover, systematic surveillance for EV infection is not routinely performed in most countries so there is incomplete understanding of the burden of disease or in trends in circulation of the > 100 different EV serotypes. NEM may be an under-recognised cause of neonatal collapse - diagnosis relies on a high index of clinical suspicion and testing for EV in suspected cases. Clinicians are reminded to consider the possibility of an underlying myocarditis and myocardial dysfunction in unwell neonates, especially in the presence of inappropriate persistent tachycardia, and to perform testing for enteroviruses as well as early cardiac investigation (electrocardiography and/or echocardiography), also detailed in our previous clinical report [11]. Of note, whole blood was the most sensitive sample type for confirming the diagnosis in this cohort. Moreover, in six cases, we documented prolonged PCR positivity in whole blood. This suggests whole blood may be useful in confirming a diagnosis even some weeks after first presentation, whereas other available clinical samples (e.g. upper respiratory tract, stool, CSF) were less frequently and more intermittently positive. Developing validated EV-PCR testing of whole blood may improve ante-mortem diagnosis of NEM, which otherwise may only be diagnosed post-mortem or post-transplant.

Treatment of severe EV infection has been primarily supportive, usually in critical care settings, though intravenous immunoglobulin (IVIg), steroids, investigational antivirals (e.g. pocapavir) and immunomodulation (e.g. anakinra) have been used [11,28,29]. Cardiopulmonary support with extra-corporeal membranous oxygenation has been used in some NEM cases but with high mortality and complication rates [8,30]. Cases of NEM should be reported to relevant public health bodies and isolates submitted to reference laboratories for confirmation, serotyping and further characterisation. Improving EV surveillance can further increase understanding about EV infection outcomes and may be useful to provide an early signal for surges in those strains known to be associated with more severe phenotypes or the emergence of new EV strains. Temporal trends in myocarditis hospitalisations can also provide useful data to consider alongside virological data. However, as hospital admission-based surveillance systems may have a significant lag, clinical surveillance is critical. Awareness among clinicians coupled with ongoing epidemiological and virological surveillance will be important for the 2023/2024 season and beyond.

## Supplementary Data

519000 Supplementary Material
