# Supplementary material for: Increased reports of severe myocarditis associated with enterovirus infection in neonates, United Kingdom, 27 June 2022 to 26 April 2023
Source: Euro Surveill. 2023 Sep 28;28(39):2300313. doi: 10.2807/1560-7917.ES.2023.28.39.2300313 (PMC10540513; doi:10.2807/1560-7917.ES.2023.28.39.2300313)
Supplement: Supplementary Material [file 23-00313_SINGANAYAGAM_Supplement.pdf]

Supplementary Information

This supplementary material is hosted by *Eurosurveillance* as supporting information alongside the article "Increased reports of severe myocarditis associated with enterovirus infection in neonates, United Kingdom, 27 June 2022 to 26 April 2023", on behalf of the authors, who remain responsible for the accuracy and appropriateness of the content. The same standards for ethics, copyright, attributions and permissions as for the article apply. Supplements are not edited by *Eurosurveillance* and the journal is not responsible for the maintenance of any links or email addresses provided therein

Contents

Supplementary Table 1: Clinical and demographic features of n=20 NEM cases from the UK, June 2022 to April 2023

Supplementary Figure 1: Total number of confirmed EV infections (all ages) in England reported through Second Generation Surveillance System (SGSS), or from the national reference laboratory, by month of year

Supplementary Figure 2: Enterovirus tests performed and percentage positivity in children aged <1 year, Wales, June 2014 to June 2023

Supplementary Figure 3: Phylogenetic of analysis of partial VP1 sequence of CVB3 and CVB4 samples sent to the national reference laboratory

		Number of cases (total n=20)	% of total
**Age (days) at diagnosis**	0-6<br>7-14<br>15-21<br>22-28<br>29-90	2<br>16<br>1<br>1<br>0	10<br>80<br>5<br>5<br>0
**Gender**	Female<br>Male	9<br>11	45<br>55
**Gestational Length**	Full term (37-42 weeks)<br>Under 37 weeks<br>Unknown	15<br>3<br>2	75<br>15<br>10
**Mode of delivery**	Vaginal<br>C-section<br>Unknown	8<br>5<br>7	40<br>25<br>35
**Critical care admission**	Yes<br>No	16<br>4	80<br>20
**Viral subtype**	CVB3<br>CVB4<br>CVB1<br>CVB5<br>Unknown	9<br>6<br>1<br>1<br>3	45<br>30<br>5<br>5<br>15
**Laboratory testing**	 Whole blood<br>Upper respiratory tract<br>Stool<br>Cerebrospinal fluid	**No. tested/no. positive**<br>16/16<br>8/18<br>10/17<br>7/8	**Proportion positive**<br>100<br>44<br>59<br>88

Supplementary Table 1. Clinical and demographic features of n=20 NEM cases

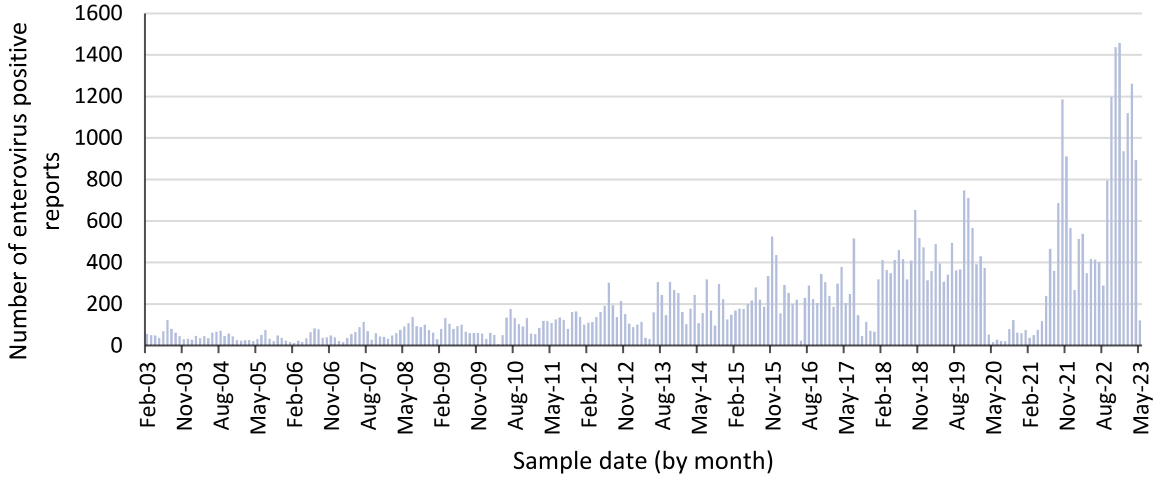

Supplementary Figure 1. Total number of confirmed EV infections (all ages) in England reported through Second Generation Surveillance System (SGSS), or from the national reference laboratory, by month of year.

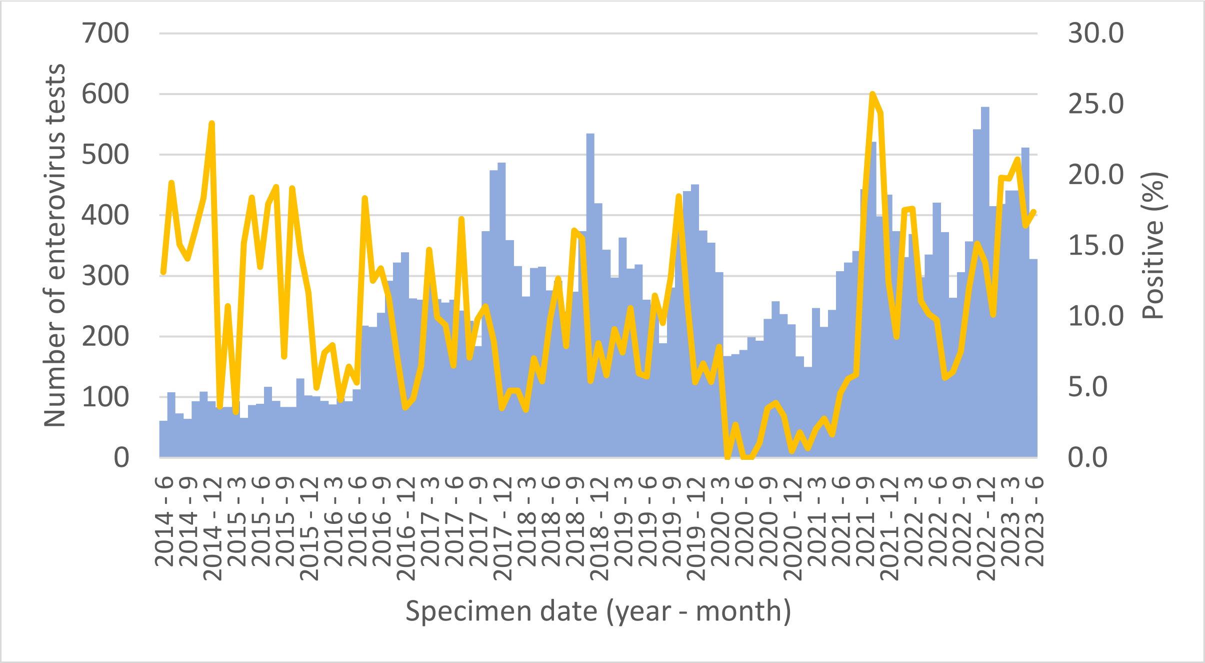

Supplementary Figure 2 – Enterovirus tests performed (blue bars) and percentage positivity (yellow line) in children aged <1 year, Wales, June 2014 to June 2023

A

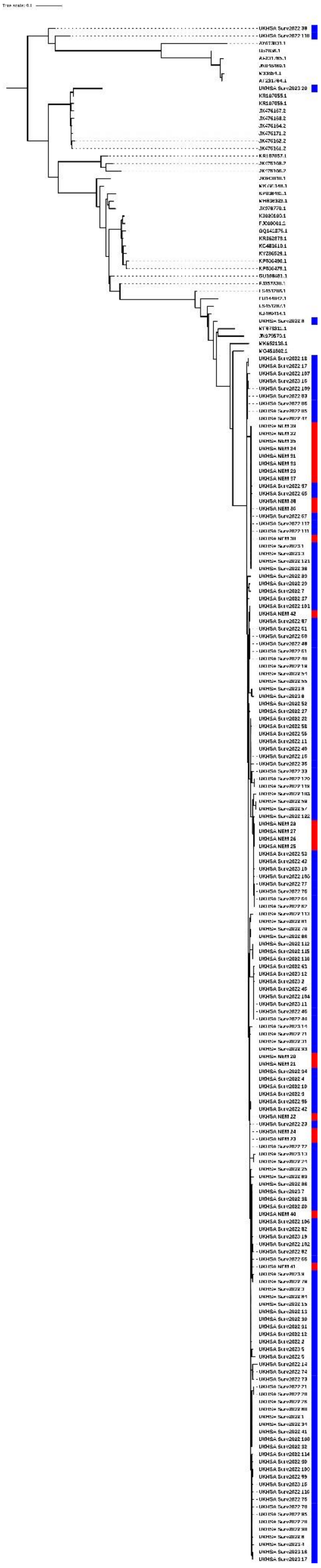

B

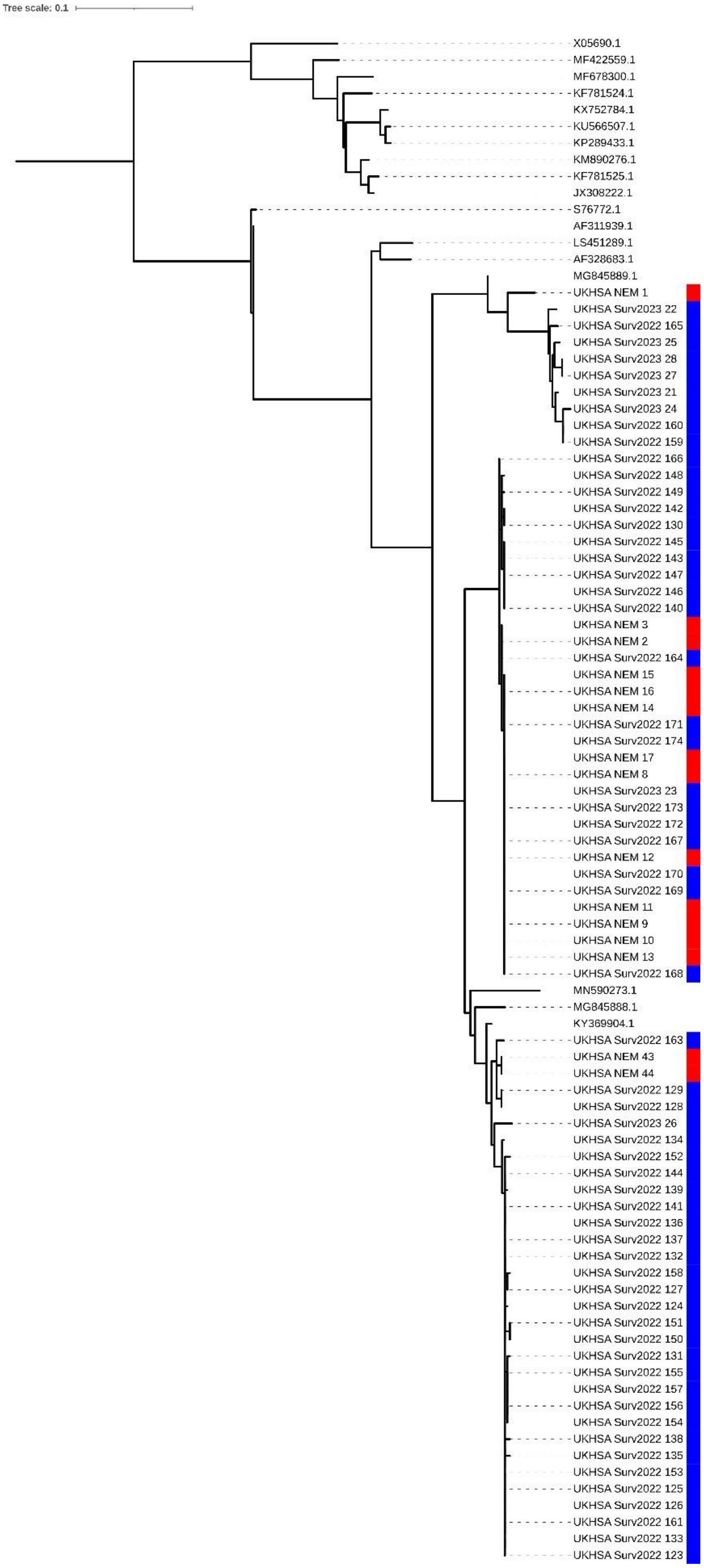

Supplementary Figure 3. Phylogenetic analysis of partial VP1 sequence from A) CVB3 - n=23 samples from NEM cases (red) and n=142 UK surveillance samples (blue) and B) CVB4 – n=15 samples from NEM cases (red) and n=59 UK surveillance samples (blue)
